# Remote Home-Based Virtual Training of Functional Living Skills for Adolescents and Young Adults With Intellectual Disability: Feasibility and Preliminary Results

**DOI:** 10.3389/fpsyg.2018.01730

**Published:** 2018-09-19

**Authors:** Simonetta Panerai, Valentina Catania, Francesco Rundo, Raffaele Ferri

**Affiliations:** ^1^Unit of Psychology, Oasi Research Institute – IRCCS, Troina, Italy; ^2^Unit of Neurology, Oasi Research Institute – IRCCS, Troina, Italy

**Keywords:** virtual reality, functional living skills, intellectual disability, remote rehabilitation, home-based rehabilitation

## Abstract

**Background:** Virtual Reality (VR) is acquiring increasing credibility as a tool for teaching independent living skills to people with Intellectual Disability (ID). Generalization of skills acquired during VR training into real environment seems to be feasible.

**Objective:** To assess feasibility and verify effectiveness of a remote home-based rehabilitation, focused on functional living skills, for adolescents and young adults with ID, by using virtual apps installed on tablets. In particular, to assess if this tool can be managed independently, if it is enjoyable and simple to be used, and if the acquired skills can be generalized to the real environment of everyday life.

**Subjects and method:** A single group, pre- and post-test research design was used. Sixteen participants with ID were included. A digital system was arranged, with a server managing communication between the database and the apps installed on tablets. *In vivo* tests were performed before and after the eleven sessions of VR training. Satisfaction questionnaires were also administered.

**Results:** Statistically significant improvements were found between the pre- and post-*in vivo* tests, as well as between the VR training sessions, in almost all the parameters taken into account, for each app. Final questionnaires showed a good satisfaction level for both the participants and their families.

**Conclusion:** The highly technological system was managed independently by participants with ID, who found it simple to be used, useful and even fun; generalization across settings was obtained. Results obtained require to be confirmed by future controlled studies, with larger samples.

## Introduction

The extensive development of new technologies enables clinicians to offer increasingly innovative therapeutic interventions. Immersive and non-immersive (desktop) Virtual Reality (VR) are among these new technologies and their popularity is due to the possibility of creating countless functional environments similar to reality, which can be adapted to different therapeutic objectives (Wilson et al., 1997). In desktop visual display systems, the person interacts with a monitor showing three-dimensional objects and environments, as well as auditory and visual stimuli that make such environments similar to the real one.

This system is simple to use, requires limited instrumentation and a short training. Several studies have shown that VR can produce a significant clinical impact and most of them have focused on the use of VR as an assessment tool, while some others show impact on rehabilitation training of cognitive, motor, social, and daily life functions (Standen and Brown, 2005; Keshner and Weiss, 2007; Optale et al., 2010; Wuang et al., 2011; Man et al., 2012; Climent-Martínez et al., 2014; Simões et al., 2014, 2018; Chiang et al., 2017). VR is acquiring increasing credibility as a useful tool for teaching independent living skills to persons with Intellectual Disability (ID) in a safe environment; furthermore, generalization into real environments of skills acquired during VR training seems to be feasible (Cromby et al., 1996a,b; Brown et al., 1998, 1999; Standen et al., 1998, 2001; Gourlay et al., 2000; Mendozzi et al., 2000; Brooks et al., 2002; Groenewegen et al., 2008; Josman et al., 2008; Saiano et al., 2015; Yang et al., 2016; Simões et al., 2018). VR training is a method in which VR environments are used for explaining or teaching certain skills to others. The aim of our study was to assess feasibility and verify the effectiveness of a new way of teaching independent living skills at home, consisting of remotely controlling persons while they use virtual apps installed on their tablets. Before the VR training, *in vivo* tests and a single teaching session to learn the use of the tablet were carried out at the Oasi Research Institute, a Sicilian center for research and treatment of ID and Neurocognitive Disorders. In order to assess feasibility, we aimed to verify: (a) if this tool can be managed independently by people with ID, (b) if it is enjoyable and simple to be used, and (c) if the acquired skills can be generalized to real environments of everyday life.

## Materials and Methods

### Participants

Sixteen adolescents and young adults with ID who lived in their family and attended daytime services were enrolled in the study. Inclusion criteria were: diagnosis of ID according with the criteria of the Statistical Manual of Mental Disorders – fifth edition (DSM-5; American Psychiatric Association, 2013), mild to moderate ID, chronological age above 15 years, sufficient reading skills, willingness to participate to the study. Exclusion criteria were: severe ID, chronological age less than 15 years, absent reading skills. The diagnoses were made according to the DSM-5 criteria by a multidisciplinary team of the Oasi Research Institute. The characteristics of the participants are reported in **Table 1**.

**Table 1:** Characteristics of the sample of subjects enrolled in this study.

**Number of subjects**	
*Total*	16
*Males*	6
*Females*	10
**Chronological age, years**	
*Mean*	26.01
*Standard deviation*	2.21
*Range*	15–48
**Intellectual disability, number of subjects**	
*Mild*	8
*Moderate*	8
**Activity limitation and participation restriction,^∗^ mean (SD), range**	
*Self care*	1.88 (1.08), 1–4
*Domestic life*	3.25 (0.58), 2–4
*Interpersonal interactions and relationships*	2.94 (0.68), 2–4
**Comorbidity, number of subjects**	
*Autism Spectrum Disorder*	4
*Niikawa-Kuroki syndrome*	1
*Smith-Magenis syndrome*	1
*Attention Deficit/Hyperactivity Disorder*	1
*Oppositional Defiant Disorder*	2
*Epilepsy*	1
*Hemiparesis*	1
**Geographical area of residence, number of subjects**	
*Sicily, North-Eastern area*	16
**Previous experience with technological devices, number of subjects**	
*Computer and/or tablet and/or smartphone*	14

*^∗^International Classification of Functioning, Disability and Health (World Health Organization, 2003): 1, mild difficulty; 2, moderate difficulty; 3, severe difficulty; and 4, complete difficulty.*

### System and Apps Description

A technological and digital system was arranged, composed by a server managing communication between the database and the apps installed on tablets, which had to be used at home. The database was developed in PostgreSQL with an interface developed with Visual Studio. The apps were created with the software Unity 3D. Each device (tablet) was first configured via a file that was downloaded at the first start of the app, depending on the protocol (task sequence) that the participant had to perform. This file contained information defining which tasks should be performed day by day. At the end of each task execution, the results were stored locally on the device. Every time the app was closed, if the internet connection was available, data synchronization between the device and the database took place via http functions.

Four apps were developed: (1) to provide *information* (30 questions in verbal and written form, including general knowledge, personal, family, spatial and temporal orientation, and with multiple-choice answers appearing on the screen in written form); (2) taking *medicines* at appropriate times (the scene presents 5 medicine boxes placed on a kitchen table; instructions explain when each drug should be taken; the task consists in choosing by touching one medicine box, for 10 times, as a response to 10 verbal questions, presented randomly during each session); (3) preparing the *suitcase* for a weekend away from home (a single scene, with shelves containing clothes to be placed in a suitcase), and (4) shopping at the *supermarket* following a shopping list (the shopping list includes 5 products and remains available on the screen; the first scene is a kitchen, with the shopping list, money and wallet; the second scene includes a supermarket shelf with different products and a shopping cart; the third scene includes the cash counter to pay for products). The apps were built on the basis of the so-called Applied Behavior Analysis (ABA). ABA is an evidence-based best practice, including behavioral principles and procedures, forming the basis for many different treatments (Cooper et al., 2007). It is recommended for educational intervention for children with both ID and ASD. VR offers the possibility to create multiple ideal environments for teaching behavioral skills to persons with neurodevelopmental disorders. Only few studies are available in the literature about the joint use of ABA and VR; however, so far the results are promising (Lakshmiprabha et al., 2014; Morford et al., 2014). In our study, several ABA techniques were included for building the apps, namely verbal reinforcement after correct response, correction after wrong response, consisting in question repetition and use of the least-to-most prompting (up to a maximum of three prompts), task analysis and total task chaining for the apps *supermarket* and *suitcase*.

### Procedures

A single group, pre and post-test research design was used covering a total period of two weeks per subject.

*In vivo* tests were performed before (on Monday of week one) and after (on Saturday of week two) the end of VR training, in real environments arranged in our center. Families who agreed to participate in the study accompanied their children to the center and were reimbursed for the trip. Before the beginning of the training, on the same Monday of week one, all participants underwent a single teaching session on the use of the apps on tablet. This session, in which researchers used some demo-games (different from the VR training apps), was carried out to guarantee the autonomous use of the system by the participants. VR training was then carried out at home for 11 consecutive daily sessions (from Tuesday of week one to Friday of week two). If difficulties were encountered, the parents or participants could use a specifically developed chat tool for contacting staff members. At the end of the training, the Satisfaction Questionnaires (SatQ) were administered to participants and families. The SatQ, especially created by the research team to assess the participants’ satisfaction level, included, in total, 14 questions with three response options: low, moderate, high, respectively scored 0, 1, and 2. The first eight questions (maximum score 16) focused on the simplicity of the system (some examples: was it easy for you to use this system? were the instructions easy to understand? did you feel comfortable using this system?), the possibility of learning throughout the VR training, the general satisfaction, the desire to continue the experience. A total score ranging from 0 to 4 and from 13 to 16, was indicative respectively of low and high satisfaction level; from 5 to 8 and from 9 to 12, the levels were considered respectively as low-moderate and moderate-high. The last six questions (maximum score 12) focused on the technological problems encountered and negative feelings (e.g., boredom, fatigue, anger, etc). A total score from 0 to 3 was considered low, from 4 to 6 low-moderate, from 7 to 9 moderate-high, and from 10 to 12 high.

The SatQ for families included 11 questions with three response options: low, moderate, high, scored respectively 0, 1, and 2. A total score from 0 to 5 was considered to indicate a low satisfaction level, from 6 to 10 low-moderate, from 11 to 16 moderate-high, from 17 to 22 high.

Virtual sessions data were collected which referred to: number of correct responses (in the *supermarket* and *suitcase* tasks, the number of correct task steps), number of errors, number of no responses (participant not answering within 10 s), and number of prompts provided. For *in vivo* tests, only the number of correct responses and total execution time were collected.

The staff involved in the VR training consisted of a project coordinator, a neuropsychologist, and an electronic engineer. *In vivo* tests were administered by a psychologist working in the diagnostic service of the research institute, blind to the aims of the study.

### Statistical Analysis

Most variables analyzed in this study did not show a normal distribution, thus, non-parametric statistics were used. The Friedman test for repeated measures was used for analyzing changes along VR training sessions; the significance level was set at *p* < 0.05. Comparisons between the first and the second *in vivo* tests were carried out by means of the Wilcoxon test for paired datasets; the significance level was set as *p* < 0.05.

### Ethics Committee Approval

Approval was obtained from the Local Committee “Comitato Etico IRCCS Sicilia – Oasi Maria SS.” as of October 26th, 2016, ethics approval code: 2016/CE-IRCCS-OASI/3/C. All parents provided written informed consent prior to the onset of the study.

Comitato Etico IRCCS Sicilia Oasi Maria SS. is organized and operates abiding by the rules of Good Clinical Practices (GCP_ICH) and by the existing regulations on clinical experiments and institution of Ethics Committees. The principal investigators are recommended to follow the ethics principles included in the Declaration of Helsinki and the Good Clinical Practice (D.M. 15/07/1997).

## Results

**FIGURE 1:**
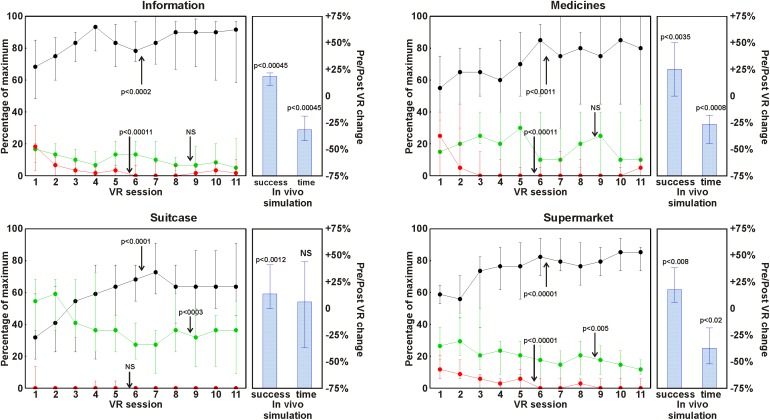
Changes along the VR training sessions in number of correct responses (black circles and lines), incorrect responses (green circles and lines), and no-responses (red circles and lines). The data are reported as a percentage of the maximum for each parameter in each app and are shown as median (circles) and interquartile range (whiskers). The histograms on the right of each line plot show the results of the *in vivo* tests expressed as a pre/post change in median percentage (columns) and interquartile range (whiskers).

Correct responses increased significantly with all apps while errors decreased in all apps, reaching statistical significance for the apps *suitcase* and *supermarket*. Also, no responses decreased in all apps, reaching statistical significance for the apps *information*, *medicines* and *supermarket.* These results are depicted in a graphical way in **Figure 1** and are reported as a percentage of the maximum for each parameter in each app. **Figure 1** does not report the number of prompts used in order to avoid too crowded graphs; however, they decreased significantly in all apps (*information, suitcase, and supermarket* *p* < 0.001, *medicines* *p* < 0.01).

Also for the *in vivo* tests, the number of correct responses increased significantly in all scenarios, while total execution time decreased significantly, with the exception of the *suitcase* task. These results are reported in **Figure 1** as a pre/post change in percentage.

With the participant SatQ (max score 16), a median score of 14 was obtained, with an interquartile range of 12–15, indicating a high level of satisfaction. The scores related to difficulties and problems encountered (max score 12) had a median of 1, with an interquartile range of 0.75–2, indicating minimal difficulties. In the family SatQ (max score 22) a median score of 15 was obtained, with an interquartile range of 11–18.25, indicating a level of satisfaction from medium to high.

## Discussion

Functional living skills are essential for people with ID for participating in family, school, community, and work life. VR is acquiring increasing credibility as a useful tool for teaching independent living skills to persons with ID in a safe environment, with important repercussion on real life. Our study assessed feasibility and verified effectiveness of a remote home-based rehabilitation, focused on functional living skills, for adolescents and young adults with ID, through VR training. In order to assess feasibility, three questions were asked of study participants: if this tool can be managed independently by people with ID, if it was simple to use, useful and even fun, and if the acquired skills could be generalized to real environments of everyday life.

After this experience we can affirm that the VR training system arranged can be managed independently by persons with mild to moderate ID and aged >15 years, without the direct control of instructors. In fact, each participant was enabled to use the system during the learning session, and all the participants used the system every day during the training phase and completed the eleven sessions (all the data was correctly recorded in the database). Furthermore, in the satisfaction questionnaire SatQ, nobody claimed need for help from others in order to use the system, nor did any family member contact the staff members to report difficulties by their children while using the system. As far as the second question is concerned, the results obtained from the SatQ clearly indicate that the participants found this approach simple to be used, useful and even fun. Finally, the skills acquired during VR training were generalized to the real environments arranged in our center, as indicated by the results from the *in vivo* tests in which also significant improvements were obtained. Therefore, it is plausible to hypothesize that they could be generalized also to real environments of everyday life.

From a clinical point of view, the most interesting advantage obtained by using this type of VR training is the positive impact on independence, shown by participants in the natural environment in the same activities carried out during the VR training. Therefore, our results confirm those of the few previous studies on the use of VR for teaching independent skills to people with ID (Cromby et al., 1996b; Brown et al., 1999; Standen et al., 2001; Brooks et al., 2002; Josman et al., 2008; Saiano et al., 2015; Yang et al., 2016). Furthermore, our data add new information, because they show that even a remote rehabilitation (without the presence of a human supervisor) enables people with ID to improve their performance in functional living skills implemented in natural environments.

However, it is essential to consider that increased independence in daily life activities is not related to a proportional reduced need for support or reduced dependence on caregivers. As shown in **Table 1**, in our sample the limitations in social participation and activities ranged from moderate to severe (in some cases complete) level, which require constant guidance from caregivers and help from support services.

While our study yields promising results, there are limitations in the research methods. We do not have a control group against which to compare our intervention group. We also note the absence of a follow-up period to measure longitudinal impact of our intervention. We urge caution in interpretation of results, and recommend future controlled studies with larger samples.

There are many advantages with using VR in teaching independent living skills. These include:

(A) The possibility to teach daily living skills also in a hospital, where domestic environments are not available, by means of virtual environments built to this purpose;
(B) Patients can be assessed and rehabilitated by performing daily living tasks in a safe, controlled, visually stimulating environment;
(C) The possibility to adapt the task difficulties to the individual characteristics of persons performing it;
(D) The possibility to carry out the sessions at any time, in the absence of a human tutor;
(E) The accuracy and completeness of data collection for monitoring the patient’s progress;
(F) The possibility to use virtual tools for the assessment and training of other functional living skills (such as, basic, home, community, school, independent, and vocational; Partington and Mueller, 2012);
(G) The satisfaction of users, who enjoy the tool;
(H) The wide applicability, potentially involving not only patients with developmental disorders, but also patients with acquired neurocognitive disorders.

Telerehabilitation provides another advantage, which is the reduction of hospital admissions, by continuing rehabilitation programs at home, with decreased rehabilitation costs and time saving for patients and their relatives (Peretti et al., 2017).

VR seems to be an approach that can be used to complement and enrich the existing rehabilitation strategies.

## Author Contributions

SP conceived the study. VC, FR, and RF contributed to the study design. SP and VC designed the apps. FR developed the apps and monitored the technological system throughout the course of the study. VC recruited participants and executed the single teaching session. SP and RF analyzed and interpreted the data. SP wrote the first version of the article. RF provided critical revisions of the manuscript. All authors have read and approved the final version of the article.

## Conflict of Interest Statement

The authors declare that the research was conducted in the absence of any commercial or financial relationships that could be construed as a potential conflict of interest.
